# Laparoscopic surgery for urachal remnants in pubescent children: a case series

**DOI:** 10.1186/s40792-020-00884-z

**Published:** 2020-06-01

**Authors:** Naoki Hashizume, Masahiro Ohtaki, Kouei Nihei, Kaoru Sakamoto, Yasuhiro Shirahata, Tetsuya Shimada, Eriko Ohta, Daisuke Yamai, Akihiro Takeshi, Kaito Sato, Satoshi Suzuki, Minoru Yagi

**Affiliations:** 1 Department of Pediatric Surgery, Tsuruoka Municipal Shonai Hospital, 4-20 Izumi-machi, Tsuruoka-shi, Yamagata, 997-0033 Japan; 2 Department of Surgery, Tsuruoka Municipal Shonai Hospital, Tsuruoka, Japan; 3 Department of Pediatric Surgery, Kurume University School of Medicine, Kurume, Japan; 4 Department of Surgery, Niigata Prefectural Tsubame Rosai Hospital, Tsubame, Japan

**Keywords:** Laparoscopic surgery, Urachal remnant, Children

## Abstract

**Background:**

Various techniques are applied in laparoscopic surgery for the treatment of urachal remnants, which are less invasive and associated with lower morbidity. We herein report a case series in which we treated urachal remnants and medial umbilical ligaments using a laparoscopic approach.

**Case presentation:**

From 2015 to 2019, seven patients (male, *n* = 5; female, *n* = 2) with a urachal remnant were treated by laparoscopic surgery in our institute. Five boys and two girls with a median age of 11 years (range 10–15 years) were enrolled in this series. The clinical results of laparoscopic treatment, the perioperative records, and the pathologic results were evaluated. The operation was performed with the use of three ports and an EZ access® (Hakko Medical, Nagano, Japan), which is a silicon cap for the wound retractor (Lap Protector®, Hakko Medical, Nagano, Japan). The removal of the urachal remnant and medial umbilical ligaments was completed with a median operative time of 92 min (range 69–128). The median hospital stay after surgery was 4 days (range 2–5). No patients developed intra-postoperative complications or recurrence.

**Conclusions:**

Although our data are preliminary, complete laparoscopic removal of symptomatic urachal remnants and medial umbilical ligaments was a safe and effective minimally invasive approach, with better cosmetic outcomes.

## Introduction

Urachal remnants occasionally require intervention when they become infected and symptomatic [1, 2]. Intervention is recommended over drainage of the abscess cavity and antibiotic therapy in order to reduce the risk of recurrence and the potential for malignant change of the urachal remnant. The traditional approach for removing a urachal remnant has been open surgery with a hypogastric transverse or midline infra-umbilical incision, which is associated with increased morbidity and longer convalescence. Recently, various techniques for applying laparoscopic surgery in the treatment of urachal remnants have been reported, which are less invasive and associated with lower morbidity [3–5]. We herein report our surgical techniques and the devices that we used in performing laparoscopic surgery for the treatment of the urachal remnant and the medial umbilical ligaments in pubescent children.

## Case presentation

### Patients

Seven patients of 10 to 15 years of age (male, *n* = 5; female, *n* = 2) with a urachal remnant were treated by laparoscopic surgery from January 2015 to December 2019. The patients’ medical records were reviewed retrospectively. All patients presented with a low abdominal infra-umbilical infection and umbilical discharge. Preoperative evaluations included ultrasonography and computerized tomography of the abdomen. Antibiotics were administered, and drainage was performed as an initial treatment. After several months of acute symptoms, each patient underwent laparoscopic excision of the urachal remnant. All values are expressed as the median (range, minimum to maximum).

### Surgical procedures

**Fig. 1:**
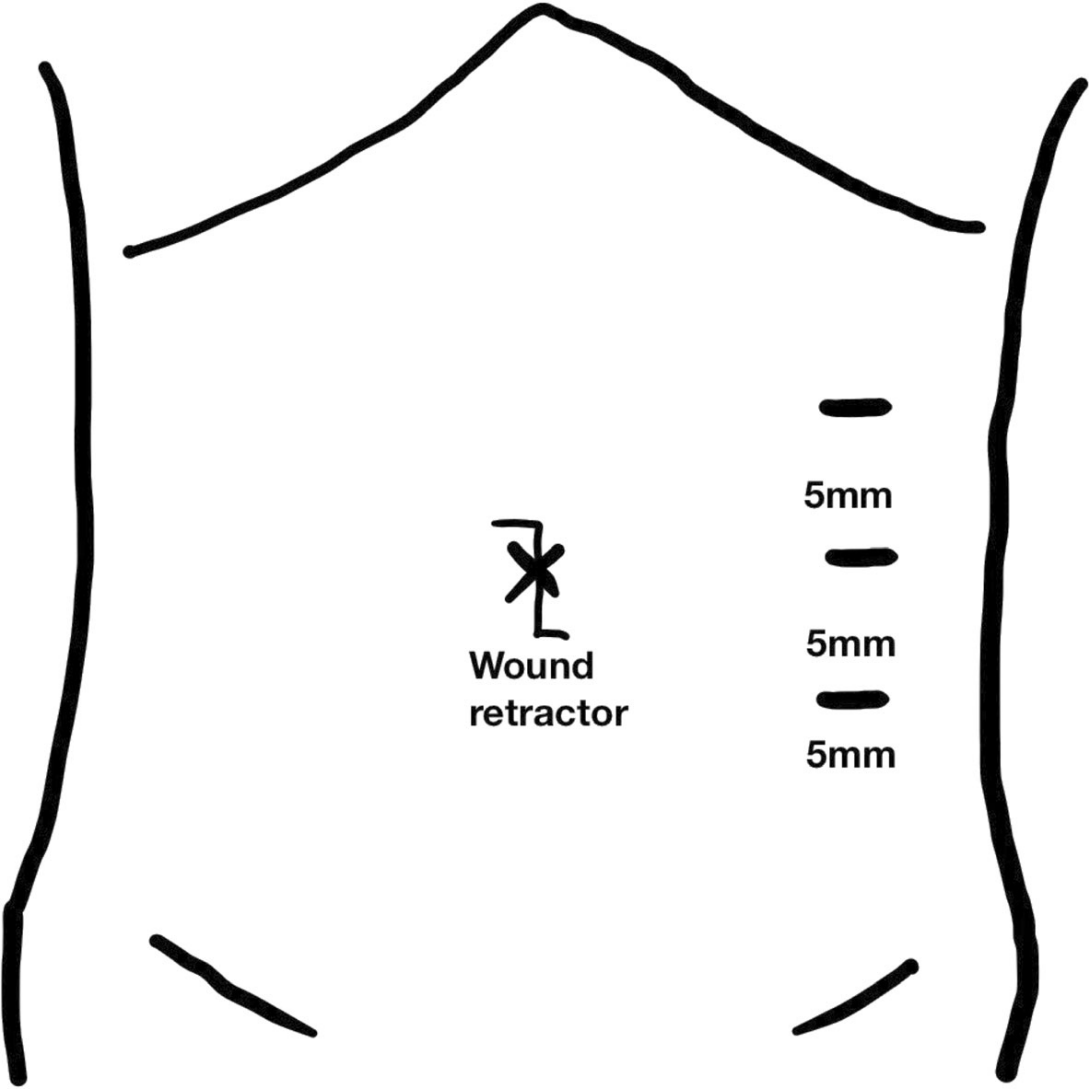
Skin incision and port placement for laparoscopic removal of a urachal remnant. After making a zigzag skin incision in the umbilicus, the fascial opening on the cephalic side of the urachus was dissected from the umbilicus. Then, on the cephalic side of the urachus and the medial umbilical ligaments, an EZ access®, which is a silicon cap for a wound retractor (Lap Protector®), was inserted intraperitoneally. Another three 5-mm ports were inserted under direct vision on the left side of the abdomen

**Fig. 2:**
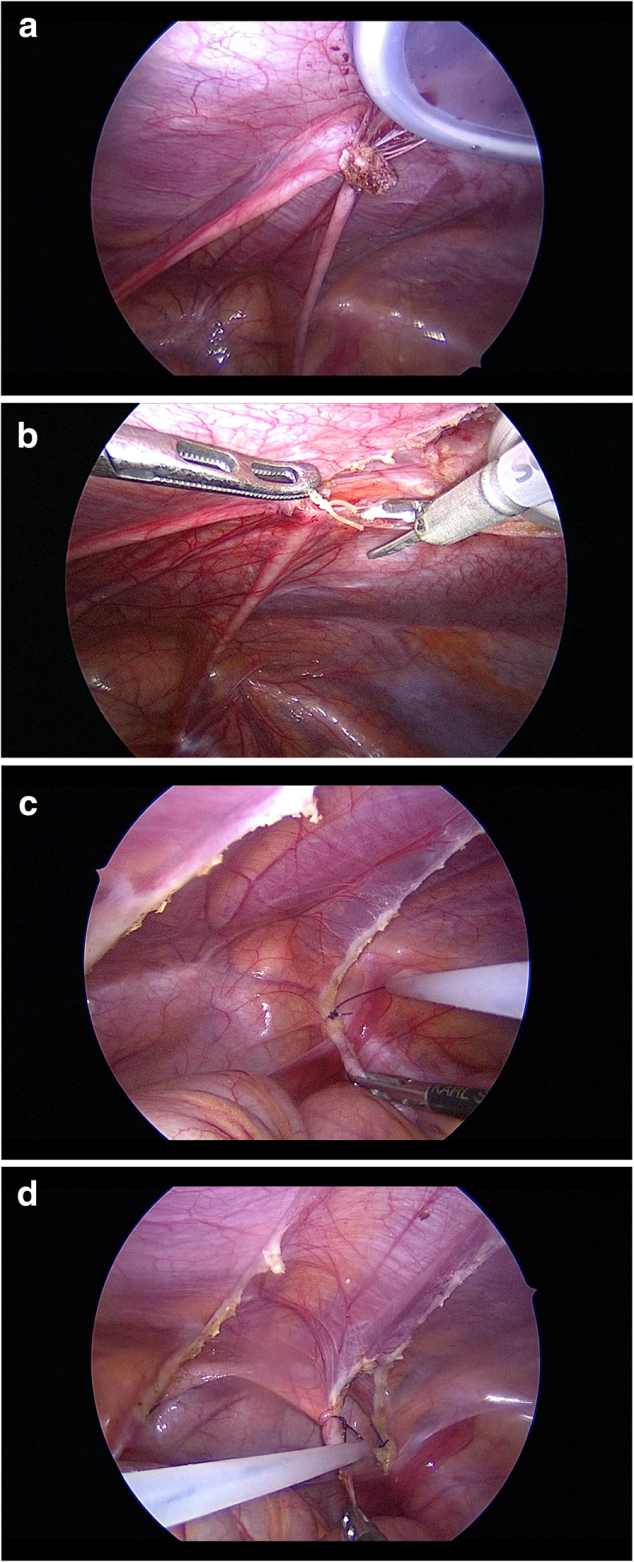
Laparoscopic view of the urachal remnant. **a** A wrap protector separated the cephalic side of the urachal remnant and the medial umbilical ligaments. **b** The urachal remnant was lysed off using ultrasonic scissors. **c** The caudal stump of the medial umbilical ligaments was tied with a single ENDOLOOP ligature®. **d** The caudal stump of the urachus was tied with a single ENDOLOOP ligature®

All patients were resected urachal remnant and the medial umbilical ligaments. Under general anesthesia, after making a zigzag skin incision at the umbilicus, the fascial opening cephalic side of the urachus was wholly dissected from the umbilicus. As medial umbilical ligaments can develop chronic infection in the event of omphalitis [6], the medial umbilical ligaments were resected continuously. After the cephalic side of the urachus and the medial umbilical ligaments were dropped into the abdominal cavity, an intraperitoneal wound retractor (Lap Protector®, Hakko Medical, Nagano, Japan) was placed in the open umbilicus. An EZ access® (Hakko Medical, Nagano, Japan), which is a silicon cap that can be used as a wound retractor with an additional 12-mm port (EZ trocar®, Hakko Medical, Japan), was placed in the cap, and insufflation was performed using CO_2_ to maintain an intraabdominal pressure of 8 mmHg. Another three 5-mm ports were inserted under direct vision on the left side of the abdomen (Fig. 1). A 30° laparoscope was used in all procedures. A wound retractor separated the dropped cephalic side of the urachus and the medial umbilical ligaments (Fig. 2a). Any bowel or omental adhesions from prior operations or inflammatory reactions of the infected urachal remnant and medial umbilical ligaments were lysed off using ultrasonic scissors (Fig. 2b). A single ENDOLOOP ligature® (ETHICON; Bridgewater, NJ, USA) was placed at the caudal stump of the medial umbilical ligaments, which was tied and then transected with ultrasonic scissors (Fig. 2c). The caudal stump of the urachus was tied with a single ENDOLOOP ligature® and transected just above the bladder dome with ultrasonic scissors (Fig. 2d). If preoperative or intraoperative investigations revealed communication or adhesion between the urachus and the bladder, we performed bladder cuff resection and then transverse suturing using a linear stapler. The defected peritoneum was not repaired. The excised specimen was exteriorized via a wound retractor and sent for a histopathological examination. The clinical results of the laparoscopic excision of urachal remnants and medial umbilical ligaments, the perioperative records, and the pathologic results were evaluated.

### Outcome

The patient demographic and perioperative data are shown in Table 1. The median patient age was 11 years (range 10–15). The median body mass index was 18.6 kg/m^2^ (range 17.1–19.1). The median interval of preoperative umbilical infection was 4 months (range 2–6). The median operative time was 98 min (range 69–128). The median hospital stay was 4 days (range 2–5). The results of the pathological evaluations were as follows: infected urachal sinus (*n* = 3), infected patent urachus and partially sealed off (*n* = 2), 1 case of a normal urachal cyst, and 1 case of a normal patent urachus that was partially sealed off. All patients started to eat an oral diet and began ambulating on postoperative day 1. In our study, there were no intraoperative complications. In addition, we found no postoperative complications, including recurrence of symptoms in patients with or without infection and intestinal obstruction for adhesion formation.

**Table 1:** Patient characteristics and perioperative data

Case	Age (year)	Sex	BMI	After infection or abscess (month)	Operation time (min)	Hospital stay (days)	Pathologic diagnosis of the urachal remnant
1	10	M	19.1	5	128	4	Infected urachal sinus
2	10	F	18.7	3	120	3	Infected patent urachus and partially seal off
3	11	M	17.4	3	92	2	Infected urachal sinus
4	11	F	18.6	4	81	3	Infected urachal sinus
5	14	M	19.1	2	93	5	Normal patent urachus and partially seal off
6	14	M	17.1	6	69	4	Normal urachal cyst
7	15	M	17.1	4	89	4	Infected patent urachus and partially seal off

## Discussion

The urachus is an embryonic connection between the bladder and allantois, which elongates as the bladder descends into the pelvis causing it to obliterate and form the medial umbilical ligament [1]. When the process of obliteration is incomplete, a remnant of the urachus will remain in either the form of a urachal cyst, patent urachus, urachal sinus, or vesicourachal diverticulum [1, 2]. An infected urachal remnant can cause abdominal pain, abdominal tenderness, fever, nausea, vomiting, dysuria, voiding difficulty, urethritis, epididymitis, and orchitis at presentation [7]. Intravenous antibiotic treatment is mandatory for infected urachal cysts. After intravenous antibiotic treatment and subsequent follow-up, surgical management has remained the standard of care, as it avoids infection and prevents malignant degeneration in later life [2].

The principal treatment for a urachal remnant is the complete excision of the whole tract. This requires a long midline skin incision in the lower abdomen, which is inevitably associated with a cosmetic disadvantage due to the conspicuous scar. To alleviate this drawback, laparoscopic excision of the urachal remnant was first demonstrated in 1993 by Trondsen et al. [8]. Since that report, there have been several trials of laparoscopic surgery to correct urachal anomalies [3–5]. It has also been claimed that a laparoscopic procedure provides better cosmesis. When the distance between the umbilicus and the dome of the urinary bladder is short, as it is in infants, the umbilical approach may be suitable for resecting a urachal remnant. The umbilical approach is appropriate for infants, and laparoscopic surgery is recommended for older children [5].

In contrast with other laparoscopic approaches to the treatment of urachal anomalies, our technique had some distinct features, including devices and trocar sites. First, the luminal wall of a urachal remnant is composed of transitional epithelium, and infection may occur due to the accumulation of materials within the cyst. In our technique, the cephalic side of the urachal remnant could be completely resected. Omphalitis secondary to symptomatic urachal remnants often necessitates simultaneous resection of the umbilicus. Since its incomplete resection can result in recurrence, adequate debridement of the infected tissue is mandatory. Many cases of laparoscopic surgery do not include simultaneous umbilical resection for symptomatic urachal remnants with open urachal resection [8, 9]. The reason for this is that umbilicoplasty is very complicated for laparoscopic surgery. This is because umbilicoplasty is very complicated for laparoscopic surgery. Our method involving en bloc resection of the umbilicus followed by umbilicoplasty was deemed the best method for achieving the complete resolution of symptoms and ensuring superior cosmesis in pubescent children. Second, another three 5-mm working camera ports were inserted under direct vision on the right side of the abdomen with the patient in a supine position. These ports provided a good view of the full length of the urachus and allowed adequate access to both the umbilicus and the bladder dome, without any hindrance or discomfort. Lateral side ports and EZ access® placements could reduce the risk of incomplete excision of the urachal remnant. Compared with other reports [10–13], the mean total operative time of this technique was shorter than that of other techniques such as multi-port technique that the first port was placed in the side of the abdomen or umbilical portion as the camera port and 2 or 3 ports inserted [10, 11] and single-site surgery technique used only EZ access® placements [12].

Third, for bladder cuff resection and subsequent transverse suturing using a linear stapler, an additional 12-mm single port was placed for the wound retractor. If severe communication or adhesion between the urachus and the bladder occurs, a wound retractor may facilitate multiple port insertion.

In our method, the defected peritoneum was not repaired. There was a controversy about suturing the defection of peritoneal layers or not. In the cesarean section, non-closure of the visceral and parietal peritoneum at lower segment cesarean section is associated with fewer postoperative complications, is more cost-effective, and is simpler than the traditional operative technique of closing both peritoneal layers [14]. In our study, there was no case of intestinal obstruction for adhesion formation.

The present study was associated with some limitations. This study was retrospective in nature and only included a small number of cases. Furthermore, there were no patients under 10 years of age.

## Conclusion

In conclusion, laparoscopic excision seems to be a safe and less invasive method for the treatment of urachal remnants. However, a prospective, large, multi-institutional randomized study is needed to validate the advantages of a laparoscopic approach to an open approach.

## Data Availability

All data generated during this study are included in this published article.

## References

[1] Aylward P, Samson K, Raynor S, Cusick R (2020). Operative management of urachal remnants: an NSQIP based study of postoperative complications. J Pediatr Surg..

[2] Stopak JK, Azarow KS, Abdessalam SF, Raynor SC, Perry DA, Cusick RA (2015). Trends in surgical management of urachal anomalies. J Pediatr Surg.

[3] Sukhotnik I, Aranovich I, Mansur B, Matter I, Kandelis Y, Halachmi S (2016). Laparoscopic surgery of urachal anomalies: a single-center experience. Isr Med Assoc J.

[4] Tatekawa Y (2019). Surgical strategy of urachal remnants in children. J Surg Case Rep.

[5] Sato H, Furuta S, Tsuji S, Kawase H, Kitagawa H (2015). The current strategy for urachal remnants. Pediatr Surg Int.

[6] Yoo KH, Lee SJ, Chang SG (2006). Treatment of infected urachal cysts. Yonsei Med J.

[7] Keingsberg K (1975). Letter: Infection of umbilical artery stimulating paret urachus. J Pediatr.

[8] Trondsen E, Reiertsen O, Rosseland AR (1993). Laparoscopic excision of urachal sinus. Eur J Surg.

[9] Jeong HJ, Han DY, Kwon WA (2013). Laparoscopic management of complicated urachal remnants. Chonnam medical journal.

[10] Tanaka K, Misawa T, Baba Y, Ohashi S, Suwa K, Ashizuka S, Yoshizawa J, Ohki T (2019). Surgical management of urachal remnants in children: open versus laparoscopic approach: a STROBE-compliant retrospective study. Medicine.

[11] Maemoto R, Matsuo S, Sugimoto S, Tokuka A (2019). Umbilical resection during laparoscopic surgery for urachal remnants. Asian journal of endoscopic surgery.

[12] Yanishi M, Kinoshita H, Matsuzaki T, Yoshida T, Ohsugi H, Taniguchi H, Sugi M, Matsuda T (2020). Laparoendoscopic single-site surgery for urachal remnants: a single-center experience. Urologia internationalis.

[13] Fujiogi M, Michihata N, Matsui H, Fushimi K, Yasunaga H, Fujishiro J (2019). Early outcomes of laparoscopic versus open surgery for urachal remnant resection in children: a retrospective analysis using a nationwide inpatient database in Japan. Journal of laparoendoscopic & advanced surgical techniques. Part A.

[14] Grundsell HS, Rizk DE, Kumar RM (1998). Randomized study of non-closure of peritoneum in lower segment cesarean section. Acta obstetricia et gynecologica Scandinavica.

